# Evaluation of a rapid immunoassay for bacteriuria in dogs

**DOI:** 10.1111/jvim.16684

**Published:** 2023-04-21

**Authors:** Craig M. Sutter, Jonathan D. Dear, Jeffrey R. Fine, Jully Pires, Jane E. Sykes, Gilad Segev, Jodi L. Westropp

**Affiliations:** ^1^ William R. Pritchard Veterinary Medical Teaching Hospital University of California, Davis Davis California USA; ^2^ Department of Veterinary Medicine and Epidemiology University of California, Davis Davis California USA; ^3^ Koret School of Veterinary Medicine The Hebrew University of Jerusalem Rehovot Israel

**Keywords:** bacteriuria, RapidBac, RIA, urinary tract infection

## Abstract

**Background:**

The ability to detect bacteriuria in dogs with a point‐of‐care test might improve medical care and antimicrobial stewardship.

**Hypothesis and Objective:**

A rapid immunoassay (RIA; RapidBac) will provide acceptable sensitivity and specificity for diagnosis of bacteriuria.

**Animals:**

Forty‐four client‐owned dogs with a clinical indication for urinalysis and aerobic bacterial urine culture.

**Methods:**

Prospective study. Urine, collected by cystocentesis, was submitted for urinalysis and culture at a diagnostic laboratory. Owners completed an enrollment questionnaire regarding their dogs' clinical signs. The RIA was performed according to the manufacturer's guidelines. Results were compared to culture.

**Results:**

Forty‐four urine specimens were evaluated from 44 dogs. The sensitivity and specificity of the RIA test to detect bacteriuria compared to urine culture were 81.8% (95% CI, 65.7%‐97.9%) and 95.5% (95% CI, 86.8%‐99.9%), respectively. For cultures yielding ≥10^3^ CFU/mL, sensitivity increased to 90.0% (95% CI, 76.9%‐100%) and specificity was similar at 95.2% (95% CI, 86.1%‐99.9%). Malodorous urine, bacteriuria, and pyuria were more likely to be present in dogs with positive RIA or urine culture results compared to dogs with negative results.

**Conclusions and Clinical Importance:**

The RIA was easy to perform and had good sensitivity and excellent specificity in this group of dogs. The RIA might be a useful screening test for decision‐making regarding antimicrobial therapy in dogs with a clinical indication for urine culture. Consideration could be given to amending the International Society for Companion Animal Infectious Disease definition of bacterial cystitis as the presence of signs of lower urinary tract disease together with positive culture or a positive RIA.

**Abbreviations**
CFU: colony forming units
ISCAID: International Society for Companion Animal Infectious Disease
LUTS: lower urinary tract signs
NPV: negative predictive value
PPV: positive predictive value
RIA: rapid immunoassay
UA: urinalysis
UC: University of California
UTI: urinary tract infections
VMTH: Veterinary Medical Teaching Hospital

## 1 INTRODUCTION

Bacterial urinary tract infections (UTI) are diagnosed in approximately one‐third to two‐thirds of dogs evaluated for signs of lower urinary tract disease.^1^, ^2^, ^3^, ^4^ The most common clinical signs of UTI in dogs include stranguria, hematuria, and pollakiuria, although malodorous urine,^1^ peri‐genital licking^1^, ^5^ and urinary incontinence^5^, ^6^ occur in dogs with positive urine cultures. According to Antimicrobial Use Guidelines of the International Society for Companion Animal Infectious Diseases (ISCAID), aerobic bacterial urine culture is preferred to confirm the presence or absence of UTI; however, clinicians often prescribe antimicrobials before receiving the results of culture, or solely based on the presence of signs of lower urinary tract disease (LUTD).^6^

Microscopic examination of the urine sediment assists with diagnosis of UTI. Light microscopic examination of specimens stained using modified Wright's stain is more sensitive and specific than microscopic examination of unstained specimens. In 1 study, the sensitivity and specificity of sediment examination using modified Wright's stain for detection of bacteriuria is 93.2% and 99.0%, respectively when compared to urine culture results.^7^ The prevalence of positive aerobic bacterial urine culture results in dogs with an inactive urine sediment is low (3.4%), suggesting that sediment examination, which is rapid and inexpensive, should be used first to identify specimens likely to yield growth on culture.^8^ However, the sensitivity of routine sediment examination for detection of UTI is dependent on the expertise of laboratory personnel and is lower in dogs with urine specific gravity ≤1.013.^9^ In 1 study, urine sediment examinations were performed in a practice environment for 80% of dogs with LUTD, but urine was submitted for culture in only 56% of these cases.^4^ Despite this, antimicrobials were prescribed in 79% of cases on the day of the dogs’ evaluation and 36% of dogs were prescribed antimicrobials despite lack of definitive evidence of UTI.

A rapid immunoassay (RIA; RapidBac Vet, Silver Lake Research, Azuza, California), is a lateral flow immunoassay for the detection of gram positive or negative bacteria in the urine of dogs.^6^, ^10^ Results are available within 20 minutes, and when evaluating urine specimens collected primarily by cystocentesis, sensitivity and specificity were 97.4% and 98.8% in urine specimens from dogs yielding growth of ≥10^3^ CFU/mL.^10^ We hypothesized this assay will provide acceptable sensitivity and specificity for diagnosis of bacteriuria in dogs with indication for urine culture. A secondary aim was to identify relationships among urine culture results, urine sediment findings, and clinical signs.

## 2 MATERIALS AND METHODS

Dogs examined at the William R. Pritchard Veterinary Medical Teaching Hospital (VMTH) at the University of California‐Davis were enrolled in the study from October 2019 through November of 2021 if the clinician deemed a urinalysis (UA) and aerobic bacterial urine culture were indicated as suggested by published guidelines.^11^ Dogs were also included if the clinician requested urinalysis and urine culture as part of a diagnostic work‐up for systemic illness (eg, sepsis, progressive azotemia) or in dogs with comorbidities where urine culture results could alter management of the dog. Only dogs with specimens obtained by cystocentesis were included in the study. Urinalysis and sediment findings that were reported from the UC Davis laboratory, were also recorded. Owners of enrolled dogs were asked to complete a clinical sign survey (Supplementary File S1). This survey was similar to that utilized in a previous study,^12^ but 2 additional clinical signs were added for owners to evaluate: pain upon urination and the presence or absence of odor to the urine. Only records with completed questionnaires were included in the data set for statistical analyses of these variables.

The RIA was performed per the manufacturer's guidelines. Any color development, in addition to the control line, on the assay strip was reported as a positive result. For positive RIA results, investigators also determined bacterial classification (gram‐negative or gram‐indeterminate) based on color development location for the lines on the assay strip. Four investigators reviewed the RIA protocol and were involved with performing and interpreting the RIA test. Each RIA test was interpreted by 1 of these 4 investigators. A portion of each urine specimen was also immediately submitted in a sterile container to the VMTH clinical chemistry and microbiology laboratories for routine urinalysis and aerobic bacterial urine culture by trained laboratory personnel. For culture, 10 μL of urine was inoculated onto 5% defibrinated sheep blood and MacConkey agars and incubated at 35°C in room air with added 5% CO_2_. Bacteria were identified using matrix‐assisted desorption‐ionization time‐of‐flight mass spectrometry (Biotyper, Bruker Daltonics, Billerca, Massachusetts), conventional biochemical testing consisting of spot tests, tubed media, or a combination of these.

### 2.1 Sample size calculation and statistical analysis

For the sample size calculation, using an expected sensitivity and specificity of 95%,^10^ a clinically acceptable width of the 95% CIs for sensitivity and specificity to be no more than 10%, and disease prevalence of 42%, it was estimated that a minimum number of 44 urine specimens were needed. Results of the RIA were compared to quantitative aerobic bacterial urine culture results from the microbiology laboratory. Sensitivity and specificity of the RIA were reported for all urine culture results and individually for specimens with either ≥10^3^ or <10^3^ CFU/mL of bacterial growth. Sensitivity and specificity were also calculated for the dogs' clinical signs compared to the urine culture outcome. A 2 sampled *t*‐test and chi‐squared test was used to evaluate relationships between RIA test results with age, and urine sediment results, respectively. Cohen's kappa coefficient (*κ*) was calculated to assess agreements between RIA test result compared to the microbiology culture result. All statistical analyses were performed using SAS version 9.4 (SAS Institute Inc). *P* values <.05 were considered significant.

## 3 RESULTS

Forty‐four urine specimens from 44 dogs were analyzed. The median age of all dogs was 78 months (range, 2‐180 months). There were 36 females (9 intact, 27 spayed) and 8 males (1 intact, 7 neutered). Breeds included golden retriever (n = 5), Labrador retriever (n = 3), Yorkshire terrier (n = 3), pug (n = 2), mastiff (n = 2), mixed breed (n = 9), and other (n = 20, 1 of each breed). Positive urine culture was documented in 22/44 (50%) of urine specimens with 28 bacterial species identified. For 20 (90%) of 22 specimens, growth was ≥10^3^ CFU/mL and in 16 of these 20 specimens growth was ≥10^5^ CFU/mL; only 2 yielded <10^3^ CFU/mL. Six dogs lacked clinical signs of lower urinary tract disease; in these dogs, urine culture was deemed indicated because of the possibility that bacteriuria might be a component of underlying disease. Two of these 6 dogs had diabetes mellitus; 1 had concurrent septic arthritis and the other had persistent emphysematous and polypoid cystitis as detected using ultrasound examination. Two dogs had urolithiasis (1 with ureterolithiasis and 1 with cystolithiasis). There was 1 dog with progressive azotemia. The remaining dog had increased frequency of urination and a portosystemic shunt. In this dog, it was unclear whether the increased frequency of urination was associated with pollakiuria or polyuria. Urine culture yielded bacterial growth in 2 of these 6 dogs, the dog with cystolithiasis (≥10^5^ CFU/mL *Klebsiella*) and the dog with emphysematous and polypoid cystitis (≥10^5^ CFU/mL *Proteus*).

Nineteen of the 44 (43%) urine specimens had positive RIA results. Using the aerobic bacterial urine culture results from in the microbiology laboratory as a gold standard, there were 4 false negatives and 1 false positive. Compared to culture, the sensitivity and specificity of the RIA test for all 44 urine specimens and categorized by CFU/mL is listed in Table 1. The kappa agreement between the RIA results and those of culture was 0.77 (95% CI 58%‐96%) for all urine specimens and 0.85 (95% CI 69%‐1.00%) for specimens with ≥10^3^ CFU/mL bacterial counts.

**TABLE 1:** The sensitivity, specificity, and 95% confidence intervals (CI) of the RIA for detecting bacteriuria in 44 urine specimens from dogs, confirmed and stratified by urine culture results.

Variable	Cases	Sensitivity (95% CI)	Specificity (95% CI)
All cases	44	81.8% (65.7%, 97.9%)	95.5% (86.8%, 99.9%)
≥10^3^ CFU/mL	20	90.0% (76.9%, 100%)	95.2% (86.1%, 99.9%)
<10^3^ CFU/mL	2	0%	95.2% (86.1%, 99.9%)

**FIGURE 1:**
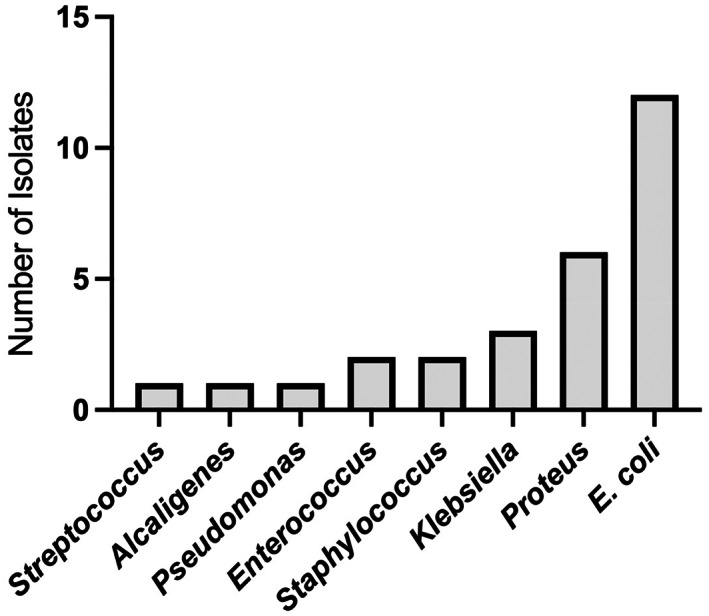
Bacteria identified from the 22 of 44 urine specimens that yielded growth on culture.

Bacteria identified by the microbiology laboratory from the 22 positive cultures are shown in Figure 1. There were 17 urine specimens with growth of a single isolate on urine culture and 5 specimens with more than 1 isolate identified. The investigators identified a gram‐negative bacterial species in 10 urine specimens using the RIA test strips, 1 of which was the single false positive. The remaining 9 true positive specimens yielded at least 1 g‐negative bacterial species on culture (*Proteus* spp. [n = 4], *Escherichia coli* [n = 3], *Klebsiella* spp. [n = 2]). In 1 of these 9 specimens both *E. coli* and *Staphylococcus* were identified. A positive RIA test that was indeterminate for gram classification was noted in specimens from another 9 dogs. Urine specimens from 6 of these dogs yielded growth of only 1 organism (*Proteus* spp. [n = 2], *E. coli* [n = 2], and *Klebsiella* spp. [n = 1]). Mixed growth was identified from the other 3 g‐indeterminate urine specimens: 1 specimen yielded growth of *Proteus* spp. and *Alcaligenes* spp., another *Proteus* spp. and *Staphylococcus* spp., and the third *Proteus* spp. and ≥10^5^ CFU/mL *Enterococcus* spp. Of the 4 false negative RIA tests, most yielded low growth on culture (10^2^ CFU/mL *Proteus* spp. [n = 1], 10^2^ CFU/mL *Pseudomonas* spp. [n = 1], 10^2^ CFU/mL *E. coli* [n = 1], 10^3^ CFU/mL *Staphylococcus* spp. [n = 1], and 10^5^ CFU/mL *Enterococcus* spp. [n = 1]). One of the false negative RIA tests was from a specimen that yielded growth of 2 species of bacteria (10^2^ CFU/mL *Pseudomonas* spp. and 10^3^ CFU/mL *Staphylococcus* spp.).

In total, 18/44 (40.9%) urine specimens had bacteriuria and 17/44 (38.6%) had pyuria (>3 WBC/hpf) on sediment analysis. Bacteriuria and pyuria were more likely to be identified on urine sediment analysis in dogs that had a positive urine culture than in dogs with a negative urine culture (81.8% vs 0%, *P* < .0001; and 72.7% vs 4.8%, *P* < .0001, respectively); similar results were obtained when evaluating bacteriuria and pyuria for a positive RIA when compared to a negative RIA result (89.5% vs 4.0%, *P* < .0001; and 68.4% vs 16.0%, *P* = .0004, respectively).

### 3.1 Clinical signs

The most common clinical sign reported by the owners for dogs was excessive licking of the dogs' peri‐genital area (n = 25; Table 2), followed by pollakiuria (n = 22), urinary incontinence (n = 16), malodorous urine (n = 12), stranguria (n = 7), hematuria (n = 8), and dysuria (n = 4).

**TABLE 2:** Summary of client assessed survey for clinical signs in 40 dogs screened for urinary tract infection.

Clinical sign	Present (N = 40)	Dogs with positive culture (n = 20)^a^	Dogs with negative culture (n = 20)^a^
Excessive licking of peri‐genital area	25 (63%)	12 (60%)	13 (65%)
Pollakiuria	22 (55%)	13 (65%)	9 (45%)
Urinary incontinence	15 (38%)	8 (40%)	7 (35%)
Malodorous urine	12 (30%)	11 (55%)	1 (5%)
Stranguria	7 (18%)	5 (25%)	2 (10%)
Hematuria	8 (20%)	5 (25%)	3 (15%)
Dysuria	4 (10%)	3 (15%)	1 (5%)

a Two missing surveys.

Completed surveys were available for 40 (91%) of 44 dogs which included 4 of the dogs that lacked clinical signs suggestive of lower urinary tract disease. Dogs with a positive urine culture were more likely to have malodorous urine noted by the owner compared to dogs with a negative urine culture (*P* = .02). No other clinical sign was associated with a positive urine culture, including pollakiuria and stranguria (*P* = .09 and .08, respectively). Of the urine specimens that were negative for bacterial growth, the most common clinical signs reported by the owners were peri‐genital licking (n = 12), urinary incontinence (n = 8), pollakiuria (n = 8), stranguria (n = 2), hematuria (n = 3), and dysuria (n = 1).

Dogs with a positive RIA were also more likely to have malodorous urine compared to dogs with a negative RIA (50.0% vs 9.1%, *P* = .006).

The sensitivity and specificity of the various clinical signs for predicting growth on urine culture is shown in Table 3. Sensitivity was generally low with the highest being associated with pollakiuria at 65.0% and peri‐genital licking at 60.0%. Specificity was generally high with hematuria, dysuria, stranguria, and malodorous urine all having a specificity ≥85.0%.

**TABLE 3:** The sensitivity, specificity, and 95% confidence intervals (CI) for individual clinical signs for predicting a positive urine culture in 40 dogs with complete questionnaires and urine culture.

Clinical sign	Sensitivity (95% CI)	Specificity (95% CI)
Hematuria	25.0% (6.0%, 44.0%)	85.0% (69.4%, 99.9%)
Dysuria	15.0% (0.0%, 30.7%)	95.0% (85.5%, 99.9%)
Pollakiuria	65.0% (44.1%, 85.9%)	55.0% (33.2%, 76.8%)
Stranguria	25.0% (6.0%, 44.0%)	90.0% (76.9%, 99.9%)
Malodorous urine	55.0% (33.2%, 76.8%)	95.0% (85.5%, 99.9%)
Peri‐genital licking	60.0% (38.5%, 81.5%)	35.0% (14.1%, 55.9%)
Urinary incontinence	40.0% (18.5%, 61.5%)	60.0% (38.5%, 81.5%)

## 4 DISCUSSION

The aim of this study to was evaluate the performance of this RIA for detection of bacteriuria in dogs with an indication for urine culture, as well as to evaluate the predictive value of clinical signs to guide further diagnostics. These data might help guide veterinarians' decision‐making regarding the submission of a urine culture to a microbiology laboratory, using the RIA test we analyzed, as well as the decision to treat the dog empirically with antimicrobials. The sensitivity and specificity of the RIA for detection of ≥10^3^ CFU/mL bacteriuria were high (90.0% and 95.2%, respectively). The *κ* coefficient for the RIA was also substantial^13^ when compared to the gold standard. Therefore, this quick, user‐friendly assay with a good sensitivity and specificity could guide veterinarians regarding their decision to treat dogs that present with clinical signs suggestive of a urinary tract infection with antimicrobials and might help improve antimicrobial stewardship. The RIA was able to accurately predict gram negative classification in specimens that yielded growth of 1 bacterial species; however, the RIA was unable to distinguish the presence of gram‐negative bacteria in polymicrobial infections or gram‐positive isolates in a single urine sample. If the RIA is positive, aerobic bacterial urine culture and antimicrobial susceptibility testing would still be recommended because the RIA does not provide any information regarding antimicrobial susceptibility. This is particularly important for those dogs with recurrent UTI or in dogs with a history of antimicrobial administration.^14^

The 90.0% sensitivity and 95.2% specificity for the RIA in our study are likely similar to previous publications (97.4% sensitivity and 98.8% specificity) for urine cultures with growth ≥10^3^ CFU/mL, and for voided specimens in dogs with LUTS (89% sensitivity and 100% specificity).^6^, ^10^ While confidence intervals in our study were larger than anticipated for evaluation of the sensitivity of this test, they were narrower than those reported by Grant et al.^6^ No confidence intervals were provided for the other study for comparison.^10^ Jacob et al^10^ scored and photographed all results and data were interpreted by 1 investigator, our study mimics how the test would be performed in most veterinary practices where the result would be interpreted at the point‐of‐care.

The (*κ*) agreement between the RIA and culture for specimens that yielded ≥10^3^ CFU/mL was higher than in the study by Jacob et al (*κ* = 0.85; strong, compared to *κ* = 0.72; moderate) and similar to the study that assessed voided urine specimens (*κ* = 0.90).^6^, ^10^ In addition, the confidence intervals from our study and those reported by Jacob et al^10^ overlapped considerably suggesting the agreements are likely very similar. This rapid RIA test could help improve antimicrobial stewardship by providing reliable results before a pet owner leaves the veterinary clinic. A negative RIA test provides evidence that antimicrobial administration might not be warranted. False negative RIA tests were uncommon. A positive RIA confirms the presence of bacteriuria, but the clinician must then decide if that is associated with UTI or if it represents subclinical bacteriuria.^11^ With a sensitive and specific assay such as this RIA, consideration could be given to updating the ISCAID guidelines definition of UTI to include a positive RIA together with the presence of LUTS.

The prevalence of positive urine cultures in our study (45% ≥ 10^3^ CFU/mL) was higher than that reported in studies that either evaluated this RIA or other point‐of‐care assays.^6^, ^10^, ^15^, ^16^ This can be attributed to our inclusion criteria, which selected for dogs that were more likely to have bacteriuria. The prevalence found in our study was similar to that found in a 2019 retrospective study that evaluated the prevalence of positive urine cultures in dogs with clinical signs of lower urinary tract disease, where 46% of the 424 specimens analyzed in dogs with such signs yielded bacterial growth.^1^

We also included urine specimens from dogs where bacteriuria might have been associated with a systemic disease process or concurrent lower urinary tract disease. Only 2 of 6 of these urine specimens yielded growth so further consideration of the circumstances when urine cultures are indicated in this set of dogs is needed. In other publications, all urine specimens analyzed by the laboratory for culture were included, and those resulting in ≥10^3^ CFU/mL were determined to be “associated with UTI”^10^ but colony counts are not predicative of UTI and dogs with ≥10^5^ CFU/mL have been reported to have subclinical bacteriuria.^17^ Only 2 specimens in our study yielded growth of <10^3^ CFU/mL, despite the presence of stranguria and perivulvar licking reported by the owners of these dogs. It is possible a low colony count UTI was present in these dogs.

Dogs with a positive aerobic bacterial urine culture or a positive RIA were significantly more likely to have pyuria or bacteriuria on urine sediment examination. In a previous study, performing urine microscopy significantly impacted the decision to treat dogs with clinical signs of lower urinary tract disease but the accuracy of microscopy for predicting UTI was only 64.5%.^4^ Not all veterinary practices perform in‐house microscopy, and isosthenuria reduces the sensitivity of this test.^9^ Furthermore, urinalysis sediment results could take hours to return from an external laboratory. Automated urine sediment instruments (eg, SediVue; IDEXX laboratories) have been introduced to veterinary practices and can facilitate rapid sediment analysis. In 1 study evaluating the SediVue analyzer in cats, the sensitivity for detection of bacteria (confirmed or suspected) in cats with a confirmed positive culture was excellent (100%) but the specificity was low (35%).^18^ To the authors' knowledge, similar studies have not yet been reported for dogs. While a urine sediment examination does provide other relevant clinical information, the RIA provides data regarding the presence or absence of bacteria within approximately 20 minutes without need for expensive equipment or advanced expertise.

Within our group of dogs with clinical signs of UTI, the only clinical sign that was positively associated with a positive culture and RIA was malodorous urine. However, because of owner subjectivity in attention to clinical signs, these results should be interpreted with caution and comparisons with other studies can be difficult to make. In a 2019 study evaluating presence of signs of lower urinary tract disease and culture outcomes, hematuria and pollakiuria were associated with positive culture on univariate analysis. The authors of that retrospective study concluded that other variables should be included in future clinical rules to optimize the overall precision for diagnosing UTI.^1^ We asked clients to complete a clinical sign survey for their dog at study enrollment that not only included classic LUTS, but also peri‐genital licking as previous studies have indicated these clinical signs are suggestive for UTI in some dogs.^5^, ^6^ Because therapeutic outcome was not an aim of this study, we did not determine whether dogs with incontinence improved with antimicrobial therapy. Standardized questionnaires should be studied further and include response to treatment that might help inform decision‐making regarding urine testing and treatment.

A potential limitation of this study was that multiple observers analyzed the RIA test results. However, not only does this does represent what is likely to occur in most veterinary practices, but this avoids the potential pitfall of a single investigator repeatedly analyzing an RIA incorrectly. While a secondary goal was to evaluate the association between clinical signs and both RIA test and urine culture outcome, the study was only powered to determine the sensitivity and specificity of the RIA test itself. A larger prospective study evaluating clinical signs, signalment, and urine culture outcome would be needed.

Overall, this study found that the RIA has an excellent sensitivity and specificity and agreement with the laboratory for detecting bacterial growth in urine of dogs. This RIA might be useful to clinicians when determining their decision for empirical therapy while awaiting aerobic bacterial culture results.

## CONFLICT OF INTEREST DECLARATION

The rapid immunoassay was provided to the authors at no cost from Silver Lake Research. The manufacturer was not involved with study design, data interpretation or manuscript preparation. Dr Sykes is on the board of directors for Lexagene, Inc and receives honoraria from IDEXX Laboratories. Dr Westropp has received honoraria from Silver Lake Medical to provide continuing education content related to UTI, but not specifically targeted at the product we investigated in this study.

## OFF‐LABEL ANTIMICROBIAL DECLARATION

Authors declare no off‐label use of antimicrobials.

## INSTITUTIONAL ANIMAL CARE AND USE COMMITTEE (IACUC) OR OTHER APPROVAL DECLARATION

Authors declare no IACUC or other approval was needed.

## HUMAN ETHICS APPROVAL DECLARATION

Authors declare human ethics approval was not needed for this study.

## Supporting information


**Data S1:** Supporting Information
