# Supplementary material for: Evaluation of a rapid immunoassay for bacteriuria in dogs
Source: J Vet Intern Med. 2023 Apr 21;37(3):1015–20. doi: 10.1111/jvim.16684 (PMC10229336; doi:10.1111/jvim.16684)
Supplement: Supplementary file 1 — Data S1: Supporting Information [file JVIM-37-1015-s001.pdf]

Patient ID: **Date:** **Day:**

Please circle the number that corresponds best to the answer regarding your pet's urination habits.

1. **Does your dog void small amounts of urine frequently?**

0	1	2	3
Never	Approximately one time per day	Approximately 2-3 times per day	Multiple (>3) times during the day

2. **Do you see blood in your dog's urine?**

0	1	2	3
Never	Approximately one time per day	Approximately 2-3 times per day	Multiple (>3) times during the day

3. **Do you feel your dog is painful when it voids urine (e.g. does s/he cry or turn around immediately to his/her back end)?**

0	1	2	3
Never	Approximately one time per day	Approximately 2-3 times per day	Multiple (>3) times during the day

4. **Does your dog lick his/her penis/vulvar area?**

0	1	2	3
Never	Approximately one time per day	Approximately 2-3 times per day	Multiple (>3) times during the day

5. **Does your dog leak urine "unconsciously" (for example when he or she is lying down at rest or when asleep)?**

0	1	2	3
Never	Approximately one time per day	Approximately 2-3 times per day	Multiple (>3) times during the day

6. **Does your dog strain to urinate?**

0	1	2	3
Never	Approximately one time per day	Approximately 2-3 times per day	Multiple (>3) times during the day

7. **Does your dog's urine have a foul odor?**

0	1
No	Yes
